# A Practical Treatise on the Efficacy of Blood-Letting in the Epidemic Fever of Edinburgh. Illustrated by Cases and Tables Extracted from the Journals of the Queensbury-House Fever Hospital

**Published:** 1819-10-01

**Authors:** 


*A Practical Treatise on the Efficacy of Blood-letting in the Epidemic Fever of Edinburgh. Illustrated by Cases and Tables extracted from the Journals of the Queensbury-House Fever Hospital.* By BENJAMIN WELSH, M. D. Superintendant of that Institution, and Member of the Royal Medical Society of Edinburgh. One Vol. Octavo, 334 pages.

FOR some time past we have observed, with much regret, the tide of energetic, and judicious practice, which swept away the dogmas and delusions of Brunonian *debility*, overflow its proper boundaries, and threaten to overwhelm even those who gave to it the original impulse. During many years our reiterated cry was—" parce puer *stimulis!*" but now it is—" utere *loris!*" Blood-letting in fever, from being proscribed in toto, is now prescribed by some, *exclusively*, as almost the only measure which is deserving of notice, in the treatment of the disease. This is one of the attendant evils on every good. Some men, and especially young men, will always be found to push the best measures to extremes, as Polonius's players tore passion to tatters, in their attempts to excel.

Among the innumerable documents which have issued from the press, demonstrative of the eminent utility of blood letting in fever, Dr. Welsh's volume occupies a conspicuous station; but it has rarely been our lot to grieve more seriously, in the perusal of a work, than on this occasion. Dr. Welsh appears to hold many of those who differ in sentiment from himself in a very contemptible light; and this tone of supercilious expression pervades the work so universally, that every lover of truth and well-wisher of science, must lament the style in which Dr. Welsh has thought proper to convey his opinions. Can our author, or any man, expect to make proselytes by such arguments as the following:

" Were it necessary for me to explain or reconcile the very different accounts that have been given of fever at different times, or by different individuals, at the same time, I should have no scruple in referring it in a great measure to the following source:—*that each, with his head full of his favourite theory, has only been attentive to elicit answers from his patients that might illustrate his own views of the disease, or which, at least, had been suggested by them.*" P. 19.

Dr. Welsh evinces no intimate knowledge of human nature, if he thinks that such a sentence as this, will have any other effect upon those minds, whose approbation he ought to covet, than that of throwing a shade of discredit over the other parts of his volume.

What will the experienced physician think who, after having had ocular evidence of the difference of fevers, in different epidemics, shall peruse the following sarcastic remark on the accurate, the amiable, and the candid Sydenham?

"A slight inspection of the works of Sydenham, will demonstrate how effectually his theory of epidemic constitution served to *conjure up varieties* of epidemic fever, which were quite unobservable to any but himself and followers."

Our pages have lately been so much occupied with the subject of fever; and the utility of bloodletting in this disease having been demonstrated over and over, we do not deem it necessary to enter into any extensive analysis of Dr. Welsh's work. We shall, however, exhibit the conclusions that he has drawn, from which it will probably be evident, that our author has viewed the merits of venesection with the kind eye of a parent; while he has drawn a shade over the evils which result from its abuse. We say *abuse*; for certainly our own observations, and those of others, in whose judgment we confide, will not permit us to sanction or recommend such practice as the following. A man is brought into the hospital on the *sixteenth* day of the fever, with low muttering delirium, subsultus tendinum; tongue and teeth covered with a thick, black, tenacious fur; skin covered with stigmata—"pulse 108, rather contracted, pretty easily compressible;" and, *under such circumstances,* he is bled to thirty ounces instantly. On the 17th day, twenty ounces were abstracted. On the 18th day twenty ounces, and so on. [*See the last case in the work*] We say nothing of the death of this man, because we would equally condemn the practice, had he survived. In the latter case, we should only have had an example of what human nature will sometimes bear.

"*Conclusions.* 1. Copious blood-letting lessens the mortality in fever. 2. It cuts short epidemic fever. 3. Even where it does not save, it protracts life. 4. It mitigates all the uneasy sensations. 5. It relieves irregular muscular action or spasm, subsultus, and singultus. 6. It removes coma or delirium. 7. It removes ischuria, constipation and cuticular constriction. 8. It removes oppression and morbid congestion. 9. It reduces the temperature and pulse. 10. It calms the respiration. 11. It diminishes thirst, and improves the appetite. 12. It often checks nausea, retching and vomiting. 13. It assists the operation of purgatives and sometimes restrains diarrhœa. 14. It conciliates sleep to the patient 15. *It may be employed to five times the quantity believed, till very lately, not only with impunity, but great advantage.* 16. Youth is no valid objection to its use. 17. Old age is no impediment to its application. 18. It is often of the greatest benefit in the most hopeless cases. 19. *It is scarcely of less utility in long continued, than in recent disease.* 20. It alters the type of the fever to one more favourable. 21. Where it dos not cut short, it lessens the average duration, both of fever and convalescence. 22. The utility of blood-letting in fever depends almost solely on its being copiously drawn; and it is on the merits of this principle alone, and more particularly its extension to a period of the disease, and under circumstances hitherto esteemed the most unfavourable for its use, that we rest our claims of originality or distinction from the other authors who have recommended the application of the lancet in continued fever. 23. The practice of copious venesection is probably yet capable of great improvement."

To the 15th and 19th of the above conclusions, we decidedly object; and the 22d is open to animadversion, since we consider that "*copiously*" is a misprint, and should be written "*early*."

While we thus demur to the *extent* of Dr. Welsh's system of blood-letting, in fever, we profess ourselves, (though this is hardly necessary) to be the steady advocates of depletion in general. We acknowledge too, that Dr. W. has laid before the medical public, in his Appendix of cases, a corpus historicum of evidence, in favour of venesection, the strongest which perhaps has hitherto appeared on the subject. Had a milder spirit of *moderation* guided both his pen and lancet, the profession would have been more edified, and the author more commended, than they now are. Dr. Welsh may not thank us, *at present*, for our unpalatable opinion. But bitter potations are generally more salutary than sweet ones; and honest counsel, though it momentarily hurt the feelings, will, ultimately, prove beneficial to the understanding.

### *Cases in Illustration.*

Such were the simplicity and uniformity of practice in the Queensbury Hospital, that the following cases, taken at random, will afford a very good idea of the whole.

"*Case* 5. July 28th. Fourth day of fever. Robert Maclellan, ætat. 24, bell-hanger, complains of severe head-ache, vertigo, throbbing of temples, pain and oppression at præcordia; severe general pains, particularly affecting the joints of the inferior extremities; occasional nausea and rigors; countenance and respiration oppressed, face flushed, and eyes are suffused. Pulse 108, small and sharp; tongue white; bowels reported regular; skin hot; appetite bad; thirst urgent; sleeps ill.

"Has been exposed to the contagion of fever. Has used no remedies.—Emittantur e brachio sanguinis ℥ xxiv. Habeat sulphatis magnesiæ ℥ j ex aqua, Mist. salin. ammon. ℥ j subinde, decoctum avenæ pro potu.

"29th. Fifth day. A very restless night, with delirium and inclination to leave his bed; still incoherent; countenance is oppressed, eye dull, and of unmeaning expression. Reports head-ache and general pains to continue severe; pain at præcodia to be gone. Pulse 104, sharp; tongue white but moist; three stools, fæces are scanty, watery, and of dark colour; blood sizy.—Emittantur e brachio sanguinis ℥ xij. Repetatur sulphas magnesiæ. Continuentur alia.

"*Evening.* Is delirious; complains much of head-ache; face is flushed; eyes wild and suffused. Pulse 100, full and strong; tongue white.—Vice ℥ xij, emittantur e brachio sanguinis ℥ xx, et sera nocte, si adhuc perstant delirium et capitis dolor, abradatur capillitium, applicentur hirudines xij fronti.

"30th. Sixth day. Having become sensible, and head-ache being much abated after the bleeding, the head was not shaved, nor the leeches applied; he has passed a good night, with profuse perspiration, which continues; expression of countenance greatly improved; flushing of face and suffusion of eyes gone; he is now free of ailment, and takes his food. Pulse 68, full and soft; tongue white but moist; three natural stools; blood sizy.—Continuentur medicamenta.

"31st. Seventh day. Convalescent.—Omittantur medicamenta.

"*August* 2d. Common diet.

"7th. Dismissed cured.

"*Case* 6. June 12th. Sixth day of fever. Ann Parkinson, ætat. 40, married, and has had six children, complains of head-ache, vertigo, tinnitus aurium, occasional throbbing of temples; of pain of abdomen aggravated by pressure, coughing, or full inspiration; pain of small of back, lassitude, loss of strength, and general soreness; has frequent sickness at stomach, and retching. Countenance is oppressed, face flushed, and eyes are suffused. Pulse 135, small and sharp; tongue pretty clean and moist; surface hot but soft; bowels costive; appetite bad; much thirst; sleeps ill. Ascribes ailments to contagion. Has had one purgative powder.—Emittantur e brachio sanguinis ℥ xx.

"℞. Submuriatis hydrargyri gr. x, pulveris antimonialis gr. v. M. Fiat bolus, q. p. sumendus, habeat mistur. salin. ammon. ℥ j subinde, decoctum avenæ pro potu.

"13th. Seventh day. Has passed a good night; countenance very much improved; head-ache, throbbing of temples, flushing of face, and suffusion of eyes gone; pain of back, sickness at stomach, and retching much abated Pulse 115, small; tongue foul; two stools; the first dark coloured, but the last was of natural appearance; blood very sizy.—Continuentur medicamenta.

"14th. Eighth day. Except occasional pain of abdomen, she is free of ailment. Pūlse 86, soft; tongue clean; two stools; appetite improved.

"15th. Ninth day. She is convalescent.—Omittantur medicamenta.

"18th. Common diet.

"19th. Dismissed cured."

